# Single-cell combined bioinformatics analysis: construction of immune cluster and risk prognostic model in kidney renal clear cells based on CD8^+^ T cell-associated genes

**DOI:** 10.1186/s40001-024-01689-8

**Published:** 2024-01-30

**Authors:** Haifeng Gao, Hang Sun, Aifeng He, Hui Liu, Zihang Zhang, Dongling Li, Weipu Mao, Jinke Qian

**Affiliations:** 1 Department of Urology, Binhai County People’s Hospital, Yancheng, 224000 China; 2 Department of Emergency, Binhai County People’s Hospital, Yancheng, 224000 China; 3 Department of Pathology, Binhai County People’s Hospital, Yancheng, 224000 China; 4 Department of Nephrology, Binhai County People’s Hospital, Yancheng, 224000 China; 5 Department of Urology, Zhongda Hospital Southeast University, 87 Dingjia Bridge Hunan Road, Nanjing, 210009 China

**Keywords:** CD8^+^ T-cell, KIRC, LAG3, TME, Single-cell analysis

## Abstract

**Background:**

Kidney cancer is an immunogenic solid tumor, characterized by high tumor burden and infiltration of CD8^+^ T cells. Although immunotherapy targeting the PD1/CTLA-4 axis has demonstrated excellent clinical efficacy, clinical outcomes in most patients are poor.

**Methods:**

We used the RNA sequencing data from the GEO database for KIRC GSE121636 and normal kidney tissue GSE131685, and performed single-cell analysis for cluster identification, pathway enrichment, and CD8^+^ T cell-associated gene identification. Subsequently, the significance of different CD8^+^ T-cell associated gene subtypes was elucidated by consensus clustering, pathway analysis, mutated gene analysis, and KIRC immune microenvironment analysis in the TCGA–KIRC disease cohort. Single gene analysis identified *LAG3* as the most critical CD8^+^ T-cell-associated gene and its function was verified by cell phenotype and immunohistochemistry in KIRC.

**Results:**

In the present study, CD8^+^ T-cell associated genes in KIRC were screened, including *GZMK*, *CD27*, *CCL4L2*, *FXYD2*, *LAG3*, *RGS1*, *CST7*, *DUSP4*, *CD8A*, and *TRBV20-1* and an immunological risk prognostic model was constructed (risk score =  − 0.291858656434841*GZMK − 0.192758342489394*FXYD2 + 0.625023643446193*LAG3 + 0.161324477181591*RGS1 − 0.380169045328895*DUSP4 − 0.107221347575037*TRBV20-1). *LAG3* was identified and proved as the most critical CD8^+^ T cell-associated gene in KIRC.

**Conclusion:**

We proposed and constructed an immunological risk prognostic model for CD8^+^ T cell-associated genes and identified *LAG3* as a pivotal gene for KIRC progression and CD8^+^ T-cell infiltration. The model comprehensively explained the immune microenvironment and provided novel immune-related therapeutic targets and biomarkers in KIRC.

**Supplementary Information:**

The online version contains supplementary material available at 10.1186/s40001-024-01689-8.

## Introduction

Kidney cancer is a common malignancy of the urinary system that originates from the renal tubular epithelium. The most common histological subtype of kidney cancer is renal cell carcinoma (RCC), accounting for approximately 90% [1, 2], including kidney renal clear cell carcinoma (KIRC, 70%), papillary RCC (10–15%), and chromophobe RCC (5%) [2]. The Global Cancer Statistics (2020) stated that RCC accounted for 2.2% of newly diagnosed cancers annually, of which 25 to 30% of patients were diagnosed as advanced or metastatic with a 5-year survival rate of 10%, and 20% to 30% of patients having a propensity for recurrence and metastasis after local operation [3–5].

Depending on tumor immunogenic characteristics, the systemic treatment of RCC has witnessed significant changes in the last 20 years [6]. Traditional immunotherapy was predominantly based on interferon (IFN)-α and interleukin (IL)-2; however, IFN-α exhibits poor efficacy and IL-2 displays high toxicity [7, 8]. The subsequent application of vascular endothelial growth factor (VEGF), tyrosine kinase inhibitors (TKIs), and mammalian target of rapamycin (mTOR) inhibitors has improved the efficacy and safety of RCC systemic treatment [6]. Recently, immunotherapy agents targeting programmed death-1 (PD-1)/programmed death-ligand 1 (PD-L1) axis alone or a combination with anti-cytotoxic T lymphocyte-associated protein 4 (CTLA-4) monoclonal antibodies or antiangiogenic agents has greatly expanded the systemic treatment options for RCC [6, 9].

Although the application of immune checkpoint inhibitors (ICIs) as tumor therapeutics has led to major improvements in the RCC systemic treatment, most patients fail to achieve a durable complete response (CR). This could be because RCC is significantly different from other solid tumors in immunogenic features, has a high mutational burden and CD8^+^ T cell infiltration, and is associated with poor prognosis [10, 11]. CD8^+^ T cells constitute the major anti-tumor effective cells in the tumor microenvironment (TME) and exert cytotoxic effects. However, their function is impaired by immunosuppressive cells or molecules in the TME [12]. Up-regulation of co-suppressor molecules, including PD1 and CTLA-4, on the surface of CD8^+^ T cells bind to relevant ligands, ultimately causing CD8^+^ T cell exhaustion [13, 14]. Therefore, blocking PD-1-mediated inhibitory signaling by monoclonal antibodies could reverse CD8^+^ T cell exhaustion, thereby hindering tumor progression. However, this contradicted CD8^+^ T cell characteristics in RCC [10, 11, 15]. Recent studies have reported that the timing of PD-1 inhibition could negatively affect T-cell priming and memory CD8^+^ T cell formation, thus contributing to more appropriate timings in RCC immunotherapy [16, 17].

No relevant studies are available to explore the function of CD8^+^ T cell-associated gene sets in the KIRC and its TME. The high accuracy and specificity of single-cell RNA sequencing allows analysis of the functional status of CD8^+^ T cells and the expression of its associated genes using the single-cell sequencing data of immune-infiltrated KIRC (GSE121636 [18]) and normal kidney (GSE131685 [19]). In the present study, we developed a CD8^+^ T cell-associated immunological risk prognostic model using CD8^+^ T cell-associated marker genes obtained from single-cell sequencing analysis to predict the survival status, tumor immune microenvironment, and immunotherapy responsiveness of KIRC patients, thereby providing a potential target and predictive evidence for immunotherapy.

## Materials

### Single-cell data filtration, pre-processing, and cluster identification

The single-cell sequencing data of KIRC GSE121636 [18] and normal kidney GSE131685 [19] were screened using the Gene Expression Omnibus (GEO) database (https://www.ncbi.nlm.nih.gov/geo/). See Table 1 for details. The Seurat package was used to generate objects and filter out poor-quality cells. The standard data pre-processing processes were performed and percentages of gene numbers, cell counts, and mitochondrial sequencing counts were calculated. The filtering criteria were genes with less than only three cells detected and disregarded cells with less than 200 detected gene numbers. Cells with less than 200 or more than 2500 genes detected and those with high mitochondrial content (> 10%) were filtered out as well. We scaled the UMI counts using a scale factor of 10,000 to normalize the library size effect of each cell. Following the log transformation of data, other factors, including “percent.mt,” “nCount_RNA,” and “nFeature_RNA” were corrected for variation regression using the ScaleData function in Seurat (v3.0.2). The corrected normalized data metric was applied to standard analysis. The top 50 variable genes were extracted for principal component analysis (PCA). The top 10 principal components were retained for UMAP visualization and clustering. Cell clustering was performed using the FindClusters function (resolution = 0.5) included in the Seurat R package.

### Differential expression and survival prognosis analysis

The “Survival R package” was used to analyze differential expression, overall survival (OS), disease-specific survival, and progression-free survival of CD8^+^ T cell-associated genes based on single-cell sequencing and assays. Furthermore, we established the correlation between key genes and clinicopathological features of KIRC and constructed a prognostic nomogram and calibration curve in the TCGA–KIRC cohort.

### Consensus clustering

A cluster analysis was performed using the “Consensus Cluster Plus R Package” [20] and agglomerative PAM clustering with a 1-Pearson correlation distances and resampling 80% of the samples for 10 repetitions. The optimal number of clusters was determined using the empirical cumulative distribution function plot.

### Identification of differentially expressed genes (DEGs)

Differential mRNA expression between subtypes was studied using the Limma package (version: 3.40.2). The screening criteria were adjusted as *p* < 0.05 and |fold change| > 2.

### Functional enrichment analysis and gene set enrichment analysis (GSEA)

The “clusterProfiler” package was used to evaluate the Gene Ontology (GO) and Kyoto Encyclopedia of Genes and Genomes (KEGG) analyses of different CD8^+^ T cell-associated subtypes, with *q*-value and *p*-value thresholds of < 0.05. Moreover, the difference in gene sets between high and low expression of CD8^+^ T cell-associated genes was further assessed using the gene set enrichment analysis (GSEA) software (http://www.broadinstitute.org/gsea/index.jsp).

### Somatic mutation and immune landscape analysis

The KIRC somatic mutation data were obtained from the TCGA GDC data portal, and waterfall plots were created using the “Maftools” package. The relative percentages of different immune cell types were determined using the ESTIMATE and xCell algorithms, and the relative percentages of immune cell types between the two subtypes were compared by landscape plots. Moreover, TISIDB (http://cis.hku.hk/TISIDB/index.php) was used to further evaluate the correlation between two subtypes and different immune indicators, including lymphocytes, immune inhibitors, immunostimulators, major histocompatibility (MHC) molecules, chemokines, and chemokine receptors.

### Construction of CD8^+^ T cell-associated risk signature

Regression analysis was performed using the Lasso–Cox method via the glmnet package. A tenfold cross-validation was set up to obtain the optimal model. The prognostic significance of genes involved was assessed using the Cox method, and the relationship between different risk scores and patient follow-up time, events, and changes in the expression of individual genes was analyzed.

### Cell lines and cell culture

Human renal cancer cell lines (786-O and ACHN) were cultured in Dulbecco’s modified Eagle medium (DMEM; Gibco Thermo Fisher Scientific, USA), containing 10% fetal bovine serum (Lonsera, Uruguay), and 1% penicillin–streptomycin solution (Keygen, China). All cell lines were purchased from the Shanghai Institutes for Biological Sciences and incubated in 95% humidified air at 37 °C and 5% CO_2_.

### RNA extraction and RT-PCR

The RNA was extracted using the RNA extraction kit (Takara Kusatsu, Japan), and the Hiscript II First-Strand cDNA Synthesis Kit was used to synthesize the complementary DNA (Vazyme, China). Reverse transcriptase-polymerase chain reaction (RT-PCR) was performed using the MonAmpTM SYBR Green qPCR Mix (Monad Biotech, China).

### Small interfering RNA

The small interfering RNAs (siRNAs) against the *LAG3* gene were designed and synthesized by GenePharma Co. (China).

### Cell proliferation and colony formation assays

For the cell proliferation assay, 1000 cells were seeded into 96-well plates for 0 h to 120 h, and 10 µL of the cell counting kit-8 (Keygen, China) solution was added per well. After a 2 h incubation at 37 °C, optical density at 450 nm (OD 450 nm) was measured using a microplate reader (Bio-Tek, USA). For the colony formation assay, cells were seeded into 6-well plates at a density of 1–2 × 10 [3] cells/well and incubated for 10 to 14 days at 37 °C. Next, the cells were washed using phosphate-buffered saline, fixed with 4% polyformaldehyde (Service Bio, China), and stained with 0.1% crystal violet solution (Keygen, China). Colonies containing > 50 cells were counted using the ImageJ 2X software 2.1.4.7 (Rawak Software Inc., Germany).

### Wound healing and transwell assay

For the wound healing assay, cells were inoculated into 6-well plates and treated with si-/nc-LAG3. A straight scratch was created on the plate with a sterilized needle tip when the cell density was approximately 70%. The cell wound edge was marked and imaged under a microscope at the starting time point. After 0 h to 24 h, the migrated distance was measured and the wound closure percentage was calculated. For transwell assays, cells were inoculated into a 24-well transwell cell apical chamber containing the matrix gel (BD, USA) for evaluating invasion and gel-free for migration. The bottom and upper chambers contained the RPMI medium and fetal bovine serum-free medium, respectively. Cells invading the bottom chambers were fixed with 4% polyformaldehyde, stained with 0.1% crystal violet solution, counted, and imaged under a microscope.

### Tissue samples and tissue microarrays

Formalin-fixed and paraffin-embedded prostate cancer tissue samples were collected from patients who underwent radical nephrectomy in the affiliated Zhongda Hospital of Southeast University, China, from April 2020 to November 2021. The study samples were from patients with KIRC, and the pathological diagnosis was confirmed by at least two pathologists. With the tumor as the center, normal tissues adjacent to the tumor were used as study materials, and two pairs of tissue microarrays were created with a 0.6 mm diameter.

### Immunohistochemistry

Formalin-fixed and paraffin-embedded tissue was dewaxed and dehydrated using xylene and serially-diluted ethanol. The tissue sections were incubated at 121 ℃ in an autoclave for 5 min to extract the antigen, following which these were incubated with anti-LAG3-monoclonal antibody at 4 ℃ overnight, and the bound antibody (Proteintech) was incubated at 37 ℃ for 30 min.

### Statistical analysis

The statistical analysis was performed using the R software (version 4.0.2). Multivariate Cox regression analyses were used to evaluate the prognostic significance. When *p* < 0.05 or log-rank *p* < 0.05, the difference was significant.

## Results

### Identification of CD8^+^ T cell-associated gene subgroups by single-cell analysis

**Fig. 1:**
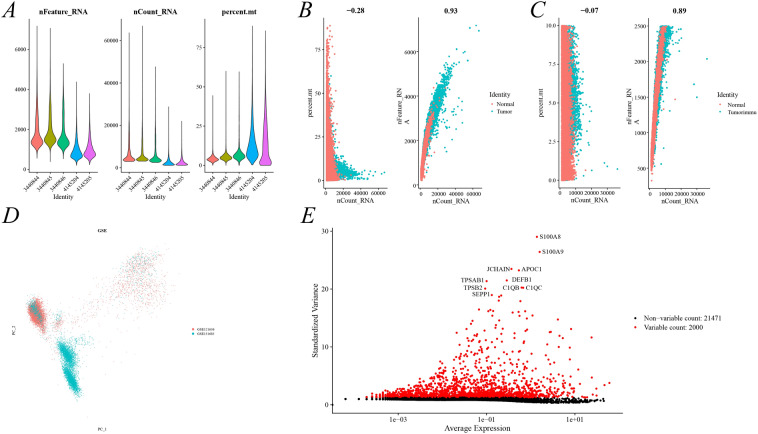
Pre-procession of single-cell sequencing data. **A** Genetic data, molecule numbers, and mitochondrial genome percentage in each cell. **B** Correlation between the primary data before filtration. **C** Correlation between primary data after filtration. **D** RPCA plot after the removal of batch effects. **E** Highly variable genes

We used the GEO database to screen a single-cell sequencing dataset about KIRC GSE121636 [18], including subsets GSE3440844, GSE3440845, and GSE3440846, as well as the normal kidney single-cell sequencing dataset GSE131685 [18], including subsets, namely, GSE4145204 and GSEE4145205. The correlation between the pre-filtered (Fig. 1B) and post-filtered (Fig. 1C) base data was analyzed by excluding cells with less than 200 or more than 2500 genes detected and cells with mitochondrial content > 10% (Fig. 1A). The analysis suggested that the cells were more highly active and that the sequencing depth was not near saturation after data filtration. Gene clusters with a high degree of variation were obtained by the PCA analysis and displayed PCA plots of PC1 and PC2 after combining to remove the batch effects (Fig. 1D), as well as obtain the top 10 highly variable features (HVGs) in 2000 HVGs including S100A8, S100A9, JCHAIN, APOC1, TPSAB1, DEFB1, TPSB2, C1QB, C1QC, and SEPP1 (Fig. 1E).

**Fig. 2:**
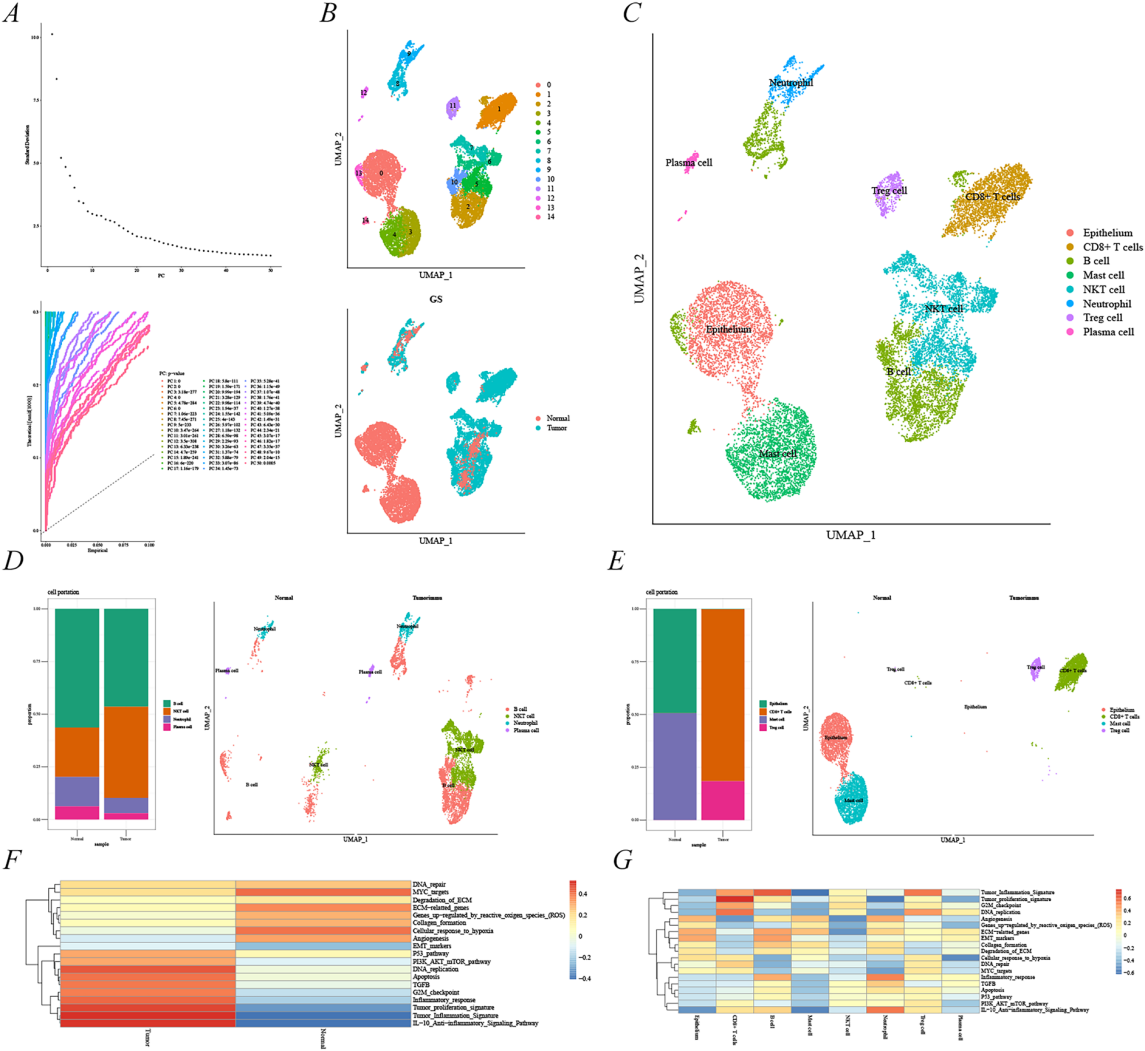
Identification of sub-groups in single-cell analysis in KIRC. **A** Determination of the principal components using ElbowPlot and JackStrawPlot() functions. **B** Cell cluster plotting using UMAP. **C** Cell cluster plotting after manual annotation of the top 10 marker genes. Plots of the distribution of shared cells (**D**) and unique cells (**E**) in normal kidney tissues and kidney cancer. **F** Metabolic pathways of normal kidney tissue and kidney cancer. **G** Metabolic pathways of different immune cells

The HVGs obtained above were included in the PCA analysis and 50 highly variable gene clusters were obtained using the ElbowPlot function and visualized by JackStrawPlot (Fig. 2A). Subsequently, KIRC cells and normal kidney tissue cells were divided into 14 clusters using the UMAP dimensionality degradation method (Fig. 2B). The top 10 genes in 14 clusters (Additional file 1: Table S1) were automatically matched to each cell type in the kidney tissue [21], ultimately obtaining eight different cell subtypes including the epithelium, CD8^+^ T cells, B cells, mast cells, NKT cells, neutrophils, Tregs, and plasma cells (Fig. 2C). A comparison of the original UMAP cluster with the artificially annotated cell cluster (Fig. 2B–C) revealed that B cells, NKT cells, neutrophils, and plasma cells were differentially expressed in KIRC and normal kidney tissue cells, with NKT cells being relatively highly expressed in KIRC (Fig. 2D). This analysis further revealed that the epithelial cells and mast cells were specific to the normal kidney tissue, whereas Tregs and CD8^+^ T cells were specific to KIRC, with CD8^+^ T cells exhibiting the highest expression (Fig. 2E). Moreover, different metabolic pathways between KIRC and normal kidney tissue were analyzed, with the KIRC tissue mostly enriched in DNA_replication, Tumor_proliferation_signature, Tumor_inflammation_signature, and IL-10_anti-inflammation_signaling_pathway, whereas the normal kidney tissue was enriched in MYC_targets, ECM-related_genes, and Cellular_response_to_hyposis (Fig. 2F). The artificially annotated cell clusters were used to study the metabolic pathways of each cell, with CD8^+^ T cell metabolic functions mostly enriched in Tumor_proliferation_signature, Tumor_inflammation_signature, G2M_checkpoint, and DNA_replication (Fig. 2G), whose metabolic functions overlapped highly with those of KIRC. The above analysis indicated that CD8^+^ T cells could be an essential immune cell population in the formation, progression, and immune response to KIRC.

### Identification of CD8^+^ T cell-associated subtypes by consensus clustering

**Fig. 3:**
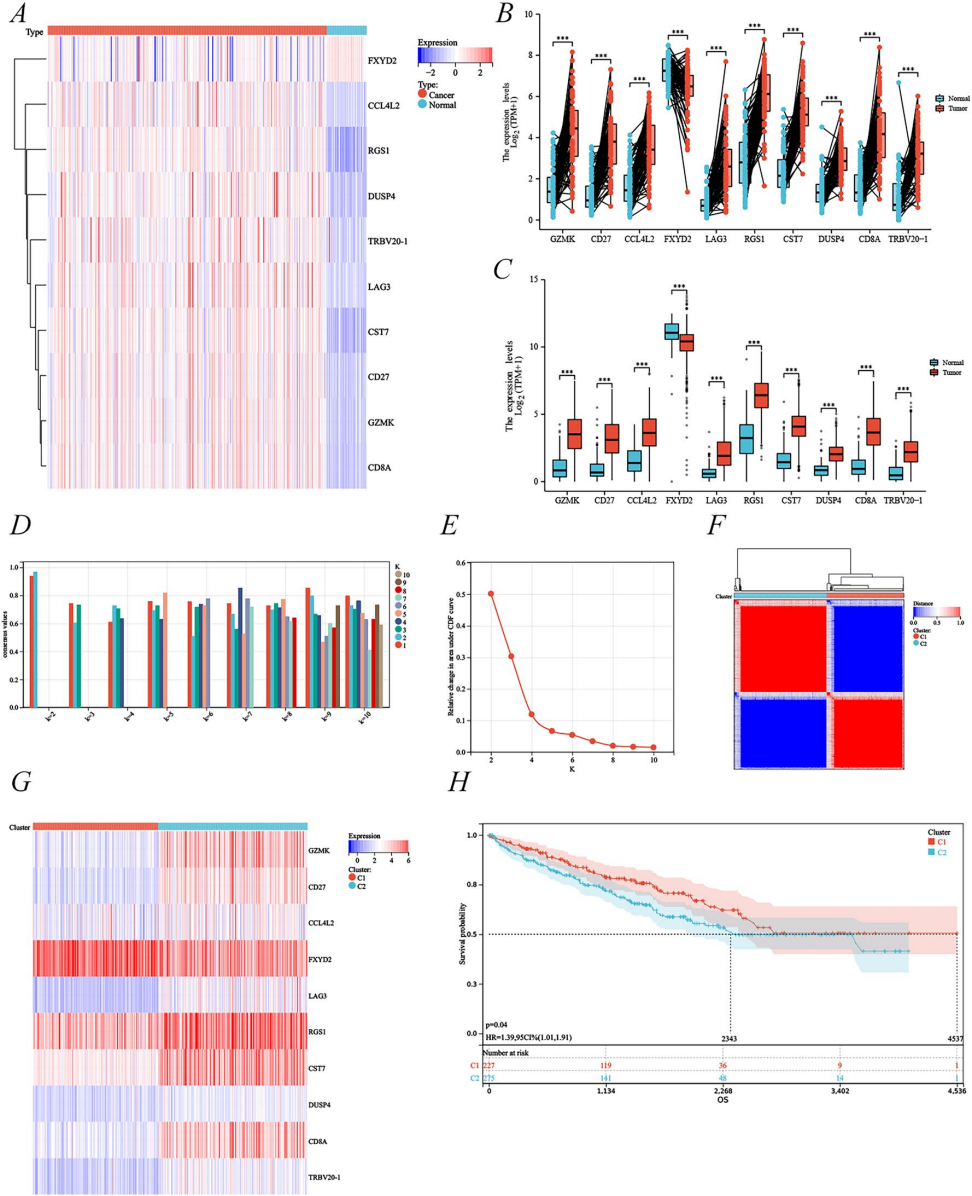
Identification of CD8^+^ T cell-associated subtypes by consensus clustering. **A** Gene expression profiles of 10 CD8^+^ T cell-associated genes in the TCGA–KIRC cohort. Paired (**B**) and unpaired (**C**) expression analyses of 10 CD8^+^ T cell-associated genes. Sample cluster consistency (**D**) and area under the distribution curve (**E**) for K from 2 to 10. **F** Consistent cluster (K = 2) of 10 genes. **G** Expression profiles of 10 CD8^+^ T cell-associated genes in different subtypes. **H** Kaplan–Meier curves for OS in C1 and C2 subtypes. C1 was a low-expression subtype of CD8^+^ T cell-associated genes and C2 was a high-expression subtype

The above single-cell sequencing analysis revealed that the top 10 CD8^+^ T cell-associated genes differentially expressed in KIRC were *GZMK*, *CD27*, *CCL4L2*, *FXYD2*, *LAG3*, *RGS1*, *CST7*, *DUSP4*, *CD8A*, and *TRBV20-1* (Additional file 1: Table S1). Subsequently, we verified the differential expression of these genes in the TCGA–KIRC cohort by heat map (Fig. 3A), paired differential expression analysis (Fig. 3B), and unpaired differential expression analysis (Fig. 3C). The results suggested that their expression was statistically significant in KIRC versus paracancerous tissues. Among these, *GZMK*, *CD27*, *CCL4L2*, *LAG3*, *RGS1*, *CST7*, *DUSP4*, *CD8A*, and *TRBV20-1* were highly expressed in KIRC and were considered oncogenes, whereas *FXYD2* was lowly expressed. The differential expression of the above genes in cancer and normal tissues was further validated using the HPA database (https://www.proteinatlas.org), which was consistent with the TCGA–KIRC cohort analysis. However, the database did not have immunohistochemical plots for CCL4L2, DUSP4, and TRBV20-1 (Additional file 3: Figure S1, Additional file 2: Table S2). Moreover, the number of clusters with the highest average consistency within the group was K = 2 (Fig. 3D), and the distribution curve was the greatest at K = 2 (Fig. 3E). Therefore, K = 2 was selected to perform clustering, and the consistent cluster (K = 2) is depicted in Fig. 3F, with 231 KIRC patients in subtype C1 and 276 KIRC patients in subtype C2. Differential expression and OS analyses of the top 10 CD8^+^ T cell-associated genes in C1 and C2 subtypes further revealed that *GZMK*, *CD27*, *CCL4L2*, *LAG3*, *RGS1*, *CST7*, *DUSP4*, *CD8A*, and *TRBV20-1* were highly expressed in the C2 subtype, whereas *FXYD2* was highly expressed in the C1 subtype (Fig. 3G). The OS was shorter in the C2 subtype compared to the C1 subtype (Fig. 3H). These results were similar to the differential expression analysis of the TCGA–KIRC cohort, indicating that the C2 subtype was the oncogenic group for KIRC.

### Identification of differentially expressed genes and signal pathways in different CD8^+^ T cell-associated subtypes

**Fig. 4:**
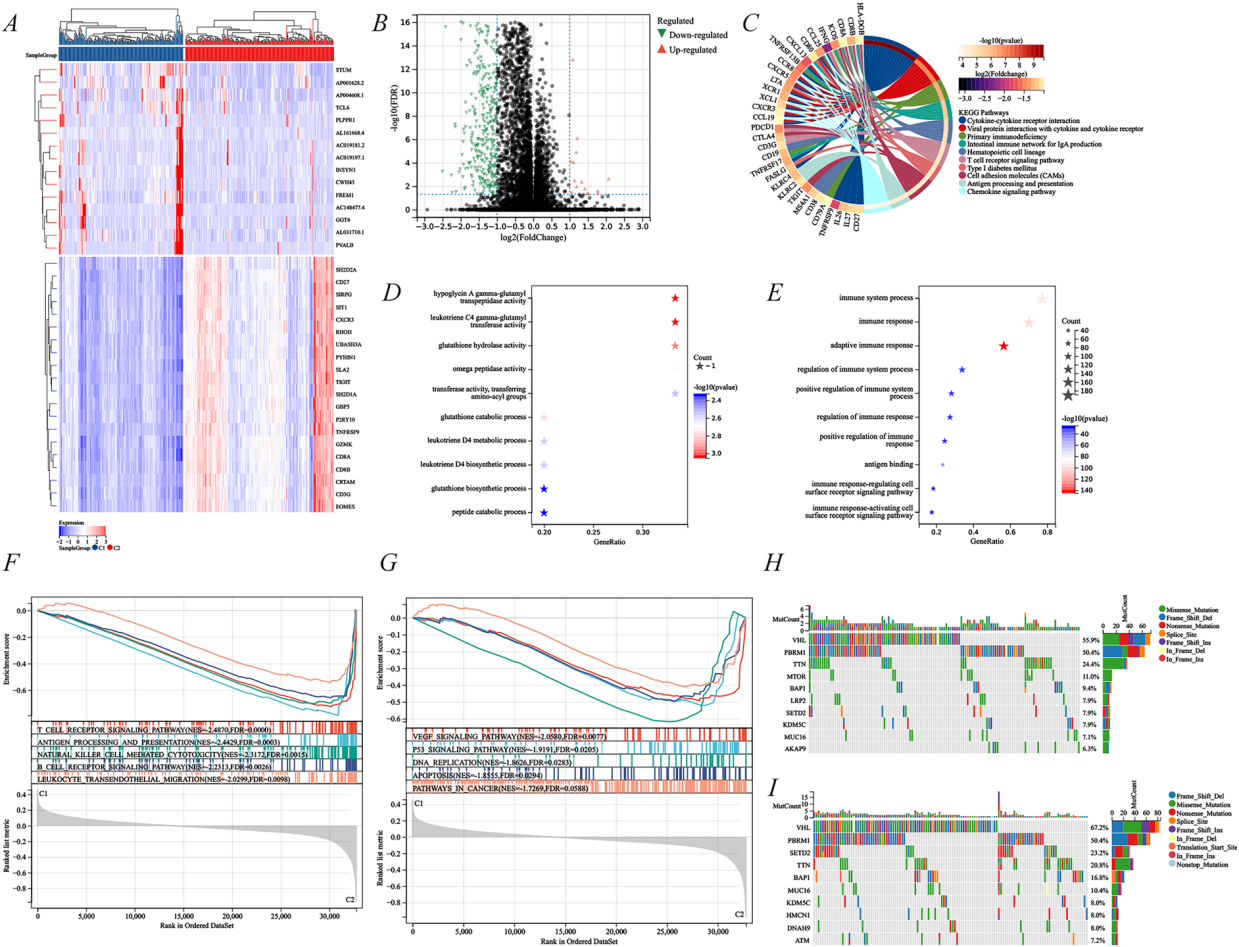
Identification of differentially expressed genes, their potential signaling pathways, and somatic mutations in C1 and C2 subtypes. DEG expression profiles (**A**) and volcano plots (**B**) of different subtypes. **C** KEGG analysis of down-regulated DEGs. GO analysis of up-regulated (**D**) and down-regulated (**E**) DEGs. Potential signaling pathways between C1 (**F**) and C2 (**G**) subtypes. Visualization of the top 10 mutated genes in the C1 (**H**) and C2 (**I**) subtypes of the CD8^+^ T cell-associated genes. C1 was a low-expression subtype of CD8^+^ T cell-associated genes and C2 was a high-expression subtype

The above analysis revealed a poor prognosis in the oncogenic subtype (C2), whereas the low expression subtype (C1) of CD8^+^ T cell-associated genes had a better prognosis. Consequently, we identified key DEGs and signaling pathways in each subtype to understand the molecular mechanisms. A total of 507 abnormally regulated genes were identified, including 15 up-regulated and 492 down-regulated genes (Fig. 4A–B). Because of a few up-regulated genes, we only performed the KEGG analysis on down-regulated genes and identified them to be mostly enriched in cytokine–cytokine receptor interaction, primary immunodeficiency, T cell receptor signaling pathway, cell adhesion molecules (CAMs), antigen processing and presentation, and chemokine signaling pathway (Fig. 4C). The up-regulated genes were largely enriched in glutamate and leukotriene activities, including hypoglycin A gamma-glutamyl transpeptidase activity, leukotriene C4 gamma-glutamyl transferase activity, and glutathione hydrolase activity (Fig. 4D). However, the down-regulated genes majorly corresponded to immune-related activities, including immune system processes, immune responses, and adaptive immune responses (Fig. 4E). Moreover, the GSEA analysis further suggested that C1 and C2 subtypes significantly differed in gene set enrichment in immune cells (T cell receptor signaling pathway, antigen processing and presentation, natural killer cell-mediated cytotoxicity, B cell receptor signaling pathway, and leukocyte transendothelial migration) (Fig. 4F) and oncogenic pathways (VEGF signaling pathway, P53 signaling pathway, DNA replication, apoptosis, and pathways in cancer) (Fig. 4G). These results suggested significant differences in gene expression and related pathway enrichment between C1 and C2 subtypes, which could be the underlying mechanism leading to the different prognosis of KIRC.

### Somatic mutations and tumor microenvironment landscape in C1 and C2 subtypes

Different somatic mutation profiles were observed between different CD8^+^ T cell-associated subtypes, and the top five genes with high mutation frequencies in the C1 subtype were VHL (55.9%), PBRM1 (50.4%), TTN (24.4%), MTOR (11.0%), and BAP1 (9.4%) (Fig. 4H). The top five genes in the C2 subtype with high mutation frequencies were VHL (67.2%), PBRM1 (50.4%), SETD2 (23.2%), TTN (20.8%), and BAP1 (16.8%) (Fig. 4I). This indicated that the mutation frequency in the oncogenic C2 subtype was higher, especially in *VHL* and *BAP1* than in the C1 subtype.

**Fig. 5:**
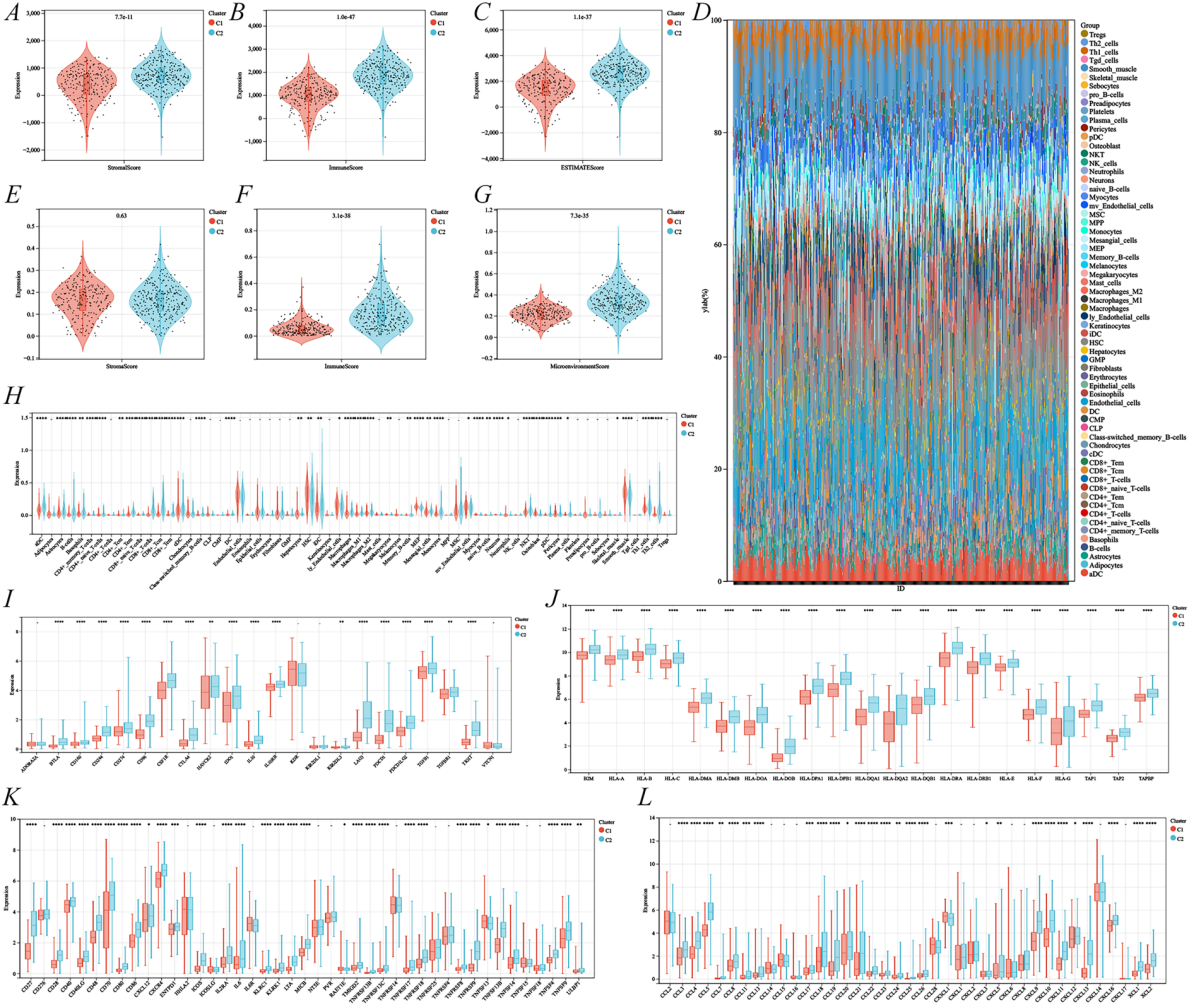
Immune landscape of C1 and C2 subtypes of CD8^+^ T cell-associated genes. Subtypes were assessed using the ESTIMATE algorithm for stromalScore (**A**), immueScore (**B**), and ESTIMATE score (**C**). Immune cell infiltration (**D**) was assessed using the xCell algorithm, as well as stromalScore (**E**), immueScore (**F**), and microenvironment Score (**G**) for different subtypes. Differential expression of genes related to immune cells (**H**), immunosuppression (**I**), MHC molecules (**J**), immune enhancement (**K**), and chemokines (**L**) between C1 and C2 subtypes. C1 was a low-expression subtype of CD8^+^ T cell-associated genes and C2 was a high-expression subtype

Because the study was based on CD8^+^ T cells, which could potentially influence the activation or silencing of tumor immune responses, we analyzed the tumor immune microenvironment in two subtypes. The StromaScore, ImmuneScore, ESTIMATEScore, and MicroenvironmentScore were higher in the C2 subtype compared to the C1 subtype (Fig. 5A–C, 5F–G) although the xCell algorithm’s StromaScore subgroup type was not meaningful (Fig. 5E). Next, the infiltration differential map of 64 immune cell types was assessed by xCell with the LM64 signature matrix (Fig. 5D). The quantitative analysis indicated that the aDC, CD8^+^ T cells, CD8^+^ Tcm, macrophages, NKTs, Th1 cells, and Th2 cells in the C2 subtype were significantly higher than those in the C1 subtype, whereas monocytes and smooth muscle cells were lower than those in the C1 subtype (Fig. 5H). Subsequently, a comprehensive analysis of the immune gene correlation between different subtypes revealed that the expression of both immune checkpoint-related genes and MHC molecules was significantly higher in the C2 subtype than in the C1 subtype (Fig. 5I–J). For immunostimulators and chemokines, the expression of CD27, CD48, ICOS, CCL5, CXCL9, and CXCL10 in the C2 subtype was significantly higher than in the C1 subtype (Fig. 5K–L).

### Construction and validation of CD8^+^ T cell-associated risk signature

**Fig. 6:**
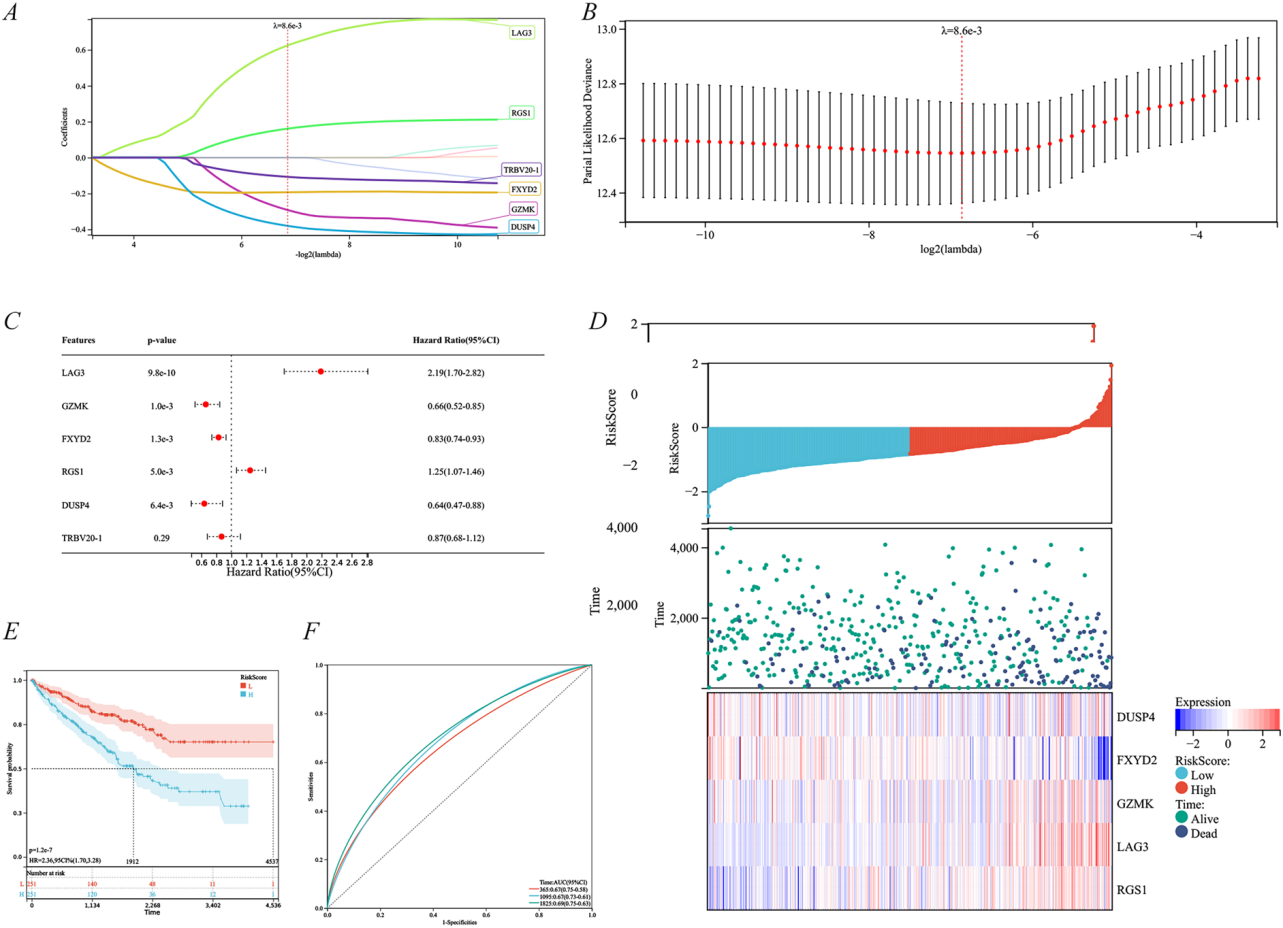
Construction and validation of CD8^+^ T cell-associated genetic risk prognostic model in the TCGA–KIRC cohort. **A–B** Correlation of 10 CD8^+^ T cell-associated genes with OS using the Lasso–Cox analysis. **C** The prognostic value of genes for OS was assessed using the univariate Cox analysis. **D** Risk score distribution, survival status, and heat map of five valuable genes. Kaplan–Meier plots (**E**) and ROC plots (**F**) of CD8^+^ T cell-associated genetic risk prognostic models

We used the LASSO regression analysis and Cox univariate analysis to construct a risk prognostic model based on CD8^+^ T cell-associated genes. After integrating survival time, survival status, and gene expression data, the final six-gene risk-score model was developed premised on 10 CD8^+^ T cell-associated genes on the LASSO regression analysis using the following model equation: RiskScore =  − 0.291858656434841*GZMK − 0.192758342489394*FXYD2 + 0.625023643446193*LAG3 + 0.161324477181591*RGS1 − 0.380169045328895*DUSP4 − 0.107221347575037*TRBV20-1 (Fig. 6A–B). Subsequently, it was finally determined by the Cox univariate analysis that GZMK, FXYD2, LAG3, RGS1, and DUSP4 displayed prognostic significance (Fig. 6C). Next, the relationship between survival status and the five genes above was investigated. Our results demonstrated that the number of survival states was considerably higher in the anti-cancer cohort (C1 subtype) compared to the oncogenic cohort (C2 subtype) (Fig. 6D). The risk scores for the above five genes were further quantified using the KM analysis in the TCGA–KIRC cohort, where high-risk scores corresponded to poorer OS (Fig. 6E) and 1, 3, and 5-year survival rates of 0.67, 0.67, and 0.69, respectively (Fig. 6F).

### Identification of LAG3 as the most critical CD8^+^ T cell-associated gene in KIRC

**Fig. 7:**
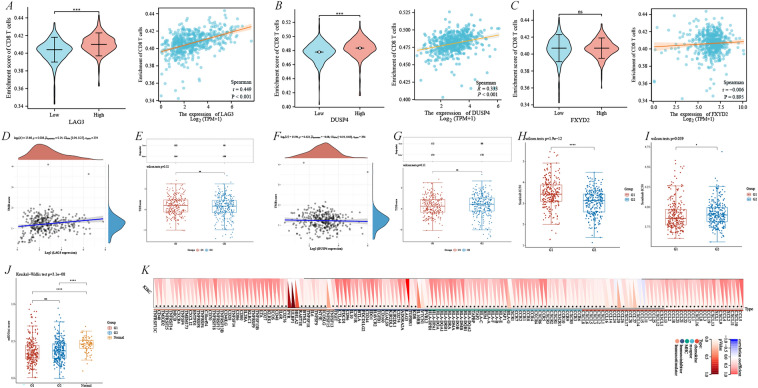
Single-gene analysis of LAG3 in CD8^+^ T cell-associated genes. The expression and correlation with CD8^+^ T cells for *LAG3* (**A**), *DUSP4* (**B**), and *FXYD2* (**C**) in the TCGA–KIRC cohort. Correlation of LAG3 with tumor mutation burden (**D**) and responsiveness to immune checkpoint inhibitors (**E**). Correlation of *DUSP4* with tumor mutation burden (**F**) and responsiveness to immune checkpoint inhibitors (**G**). IC_50_ of sunitinib (**H**) and sorafenib (**I**) in different *LAG3* expression subgroups. (**J**) *LAG3* stemness score. (**K**) Relevance of *LAG3* to immune-related genes

The correlation of CD8^+^ T cell-related genes (*GZMK*, *CD27*, *CCL4L2*, *FXYD2*, *LAG3*, *RGS1*, *CST7*, *DUSP4*, *CD8A*, and *TRBV20-1*) was further explored with prognosis and diagnosis in KIRC. We further validated the OS, disease-specific survival, progression-free interval, diagnostic receiver operating characteristic (ROC), and time-dependent ROC curves of the above 10 genes in the TCGA–KIRC cohort. The results suggested that the area under the ROC curve was > 0.85 for all genes, except *FXYD2*, and the diagnostic effect was good (Additional file 4: Figure S2A–J). LAG3 exerted a significant pro-carcinogenic effect and KIRC patients with high LAG3 expression had shorter OS (*p* = 0.008), disease-specific survival (*p* = 0.006), and progression-free interval (*p* = 0.043) (Additional file 4: Figure S2H). In contrast, *FXYD2* and *DUSP4* displayed a significant anti-carcinogenic effect with all *p* < 0.05 (Additional file 4: Figure S2E–F), whereas the other single genes exerted no great prognostic significance (Additional file 4: Figure S2). Notably, only LAG3 exhibited a relatively good time-dependent curve, AUC_1-year_ = 0.592, AUC_3-year_ = 0.574, and AUC_5-year_ = 0.580 (Additional file 4: Figure S2H). Further analysis of its association with CD8^+^ T cells revealed that the expression of *LAG3* and *DUSP4* was positively correlated with CD8^+^ T cell enrichment (Fig. 7A–B), whereas *FXYD2* was not (Fig. 7C). The correlation between key genes and immunotherapy was further explored by TMB scores, suggesting that LAG3 was positively correlated with TMB scores (ρ_Spearman_ = 0.14 and *p* = 0.006), whereas DUSP4 was insignificant (Fig. 7D, F). The study revealed that the expression of *LAG3* and *DUSP4* was not associated with TIDE scores (Fig. 7E, G). Compared to the expression of *DUSP4*, *LAG3* displayed a higher correlation with CD8^+^ T cells and a function in immunotherapy. Therefore, we followed up with an in-depth exploration of LAG3. We further verified the correlation between *LAG3* expression and different T-cell subtypes in the TCGA–KIRC disease cohort. We found that the LAG3 level was positively correlated with several T cells, including CD8 T cells, cytotoxic cells, T cells, T helper cells, Tcm, Tem, TFH, Th1 cells, Th2 cells, and Tregs, and negatively correlated with Th17 cells, with no correlation with Tgd (Additional file 5: Figure S3). Therefore, *LAG3* was identified as the most critical T cell-related prognostic gene in KIRC. Further investigation of the association of LAG3 expression with sunitinib and sorafenib sensitivity indicated that the high *LAG3* expression group (G2) had lower sunitinib IC_50_ and higher sorafenib IC_50_ (Fig. 7H–I). Moreover, *LAG3* was correlated with multiple immune-related genes (Fig. 7K). This study revealed that the *LAG3* expression was not associated with stemness scores between the high and low *LAG3* expression groups (Fig. 7J).

**Fig. 8:**
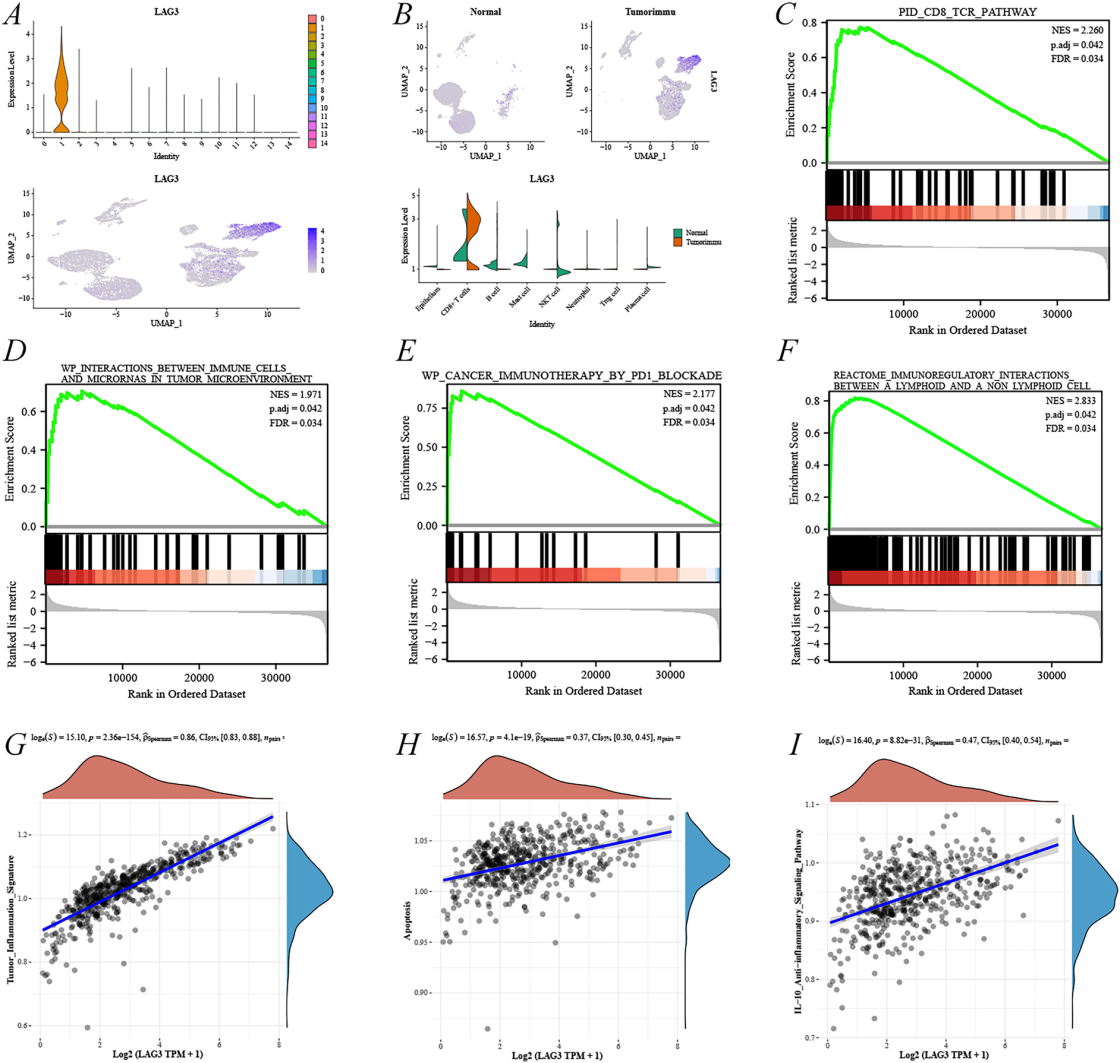
Cell population expression and pathway analysis of LAG3 in single-cell analysis. **A** LAG3 UMAP plot in CD8^+^ T cell-associated subgroups. **B** Expression of *LAG3* in different cell populations in normal kidney and renal cancer tissues. **C**–**F** Analysis of potential signaling pathway of *LAG3* by GESA. **G**–**I** Analysis of the enrichment of *LAG3* using the GSVA algorithm

The single-cell analysis revealed that *LAG3* expression was mostly concentrated in zone 1 with scattered distribution in zones 2, 5, 6, 7, and 10 (Fig. 8A). Cluster identification demonstrated that LAG3 was concentrated in CD8^+^ T cells in KIRC, whereas it was highly scattered in NKT or B cells in both normal kidney tissues and KIRC (Fig. 8B). The GSEA pathway after single-gene differential analysis suggested that its pathway included the PID_CD8_TCR_PATHWAY, WP_INTERACTIONS_BETWEEN_IMMUNE_CELLS_AND_MICRORNAS_IN_TUMOR_MICROENVIRONMENT, WP_CANCER_IMMUNOTHERAPY_BY_PD1_BLOCKADE and REACTOME_IMMUNOREGULATORY_INTERACTIONS_BETWEEN_A_LYMPHOID_AND_A_NON_LYMPHOID_CELL, whose NES > 1.9, p.adj < 0.05, and FDR < 0.05 (Fig. 8C–F). In addition, the combined enrichment scores of each sample on multiple pathways revealed the most significant positive correlation between LAG3 and Tumor_Inflammation_Signature, apoptosis, and IL-10_Anti-inflammatory_Signaling_Pathway (Fig. 8G–I).

### Validation of LAG3 as a key CD8^+^ T cell-associated gene in KIRC in in vitro experiments

**Fig. 9:**
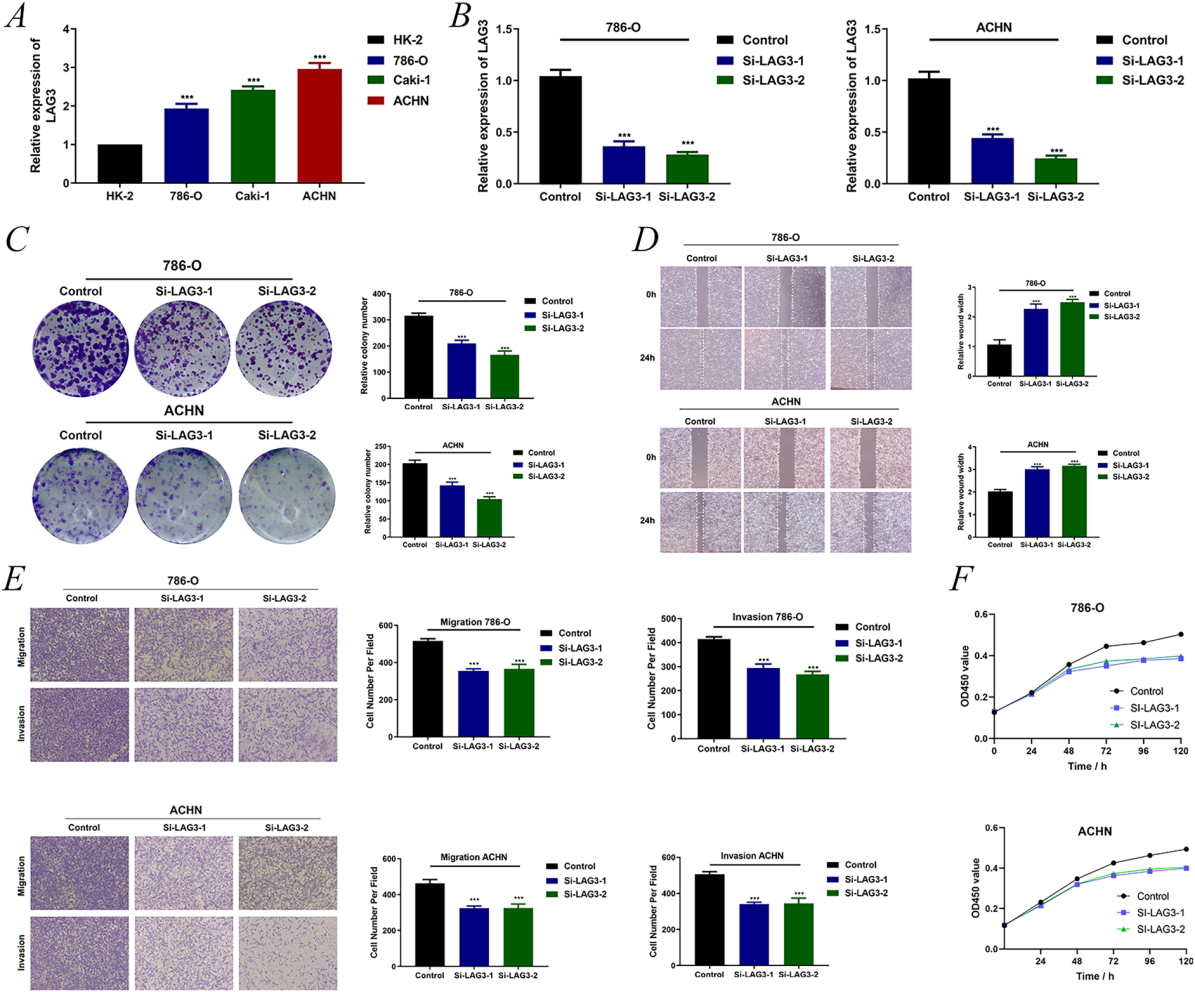
Validation of *LAG3* as a key oncogene in KIRC in in vitro experiments. **A** Validation of *LAG3* expression in renal cancer cell lines. **B** Knock-down efficiency of *LAG3*, respectively, in 786-O and ACHN cell lines. **C** LAG3 clone tests in 786-O and ACHN cell lines. **D** LAG3 scratch tests in 786-O and ACHN cell lines. **E** LAG3 migration and invasion assays in 786-O and ACHN cell lines using a 24-well plate. **F** LAG3 CCK8 assay in 786-O and ACHN cell lines

**Fig. 10:**
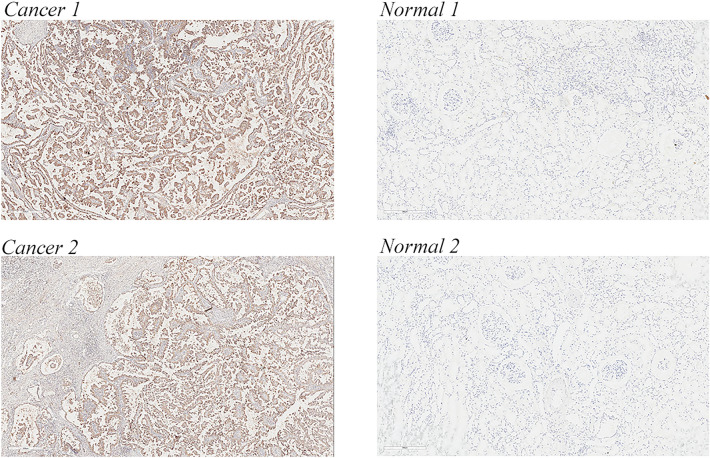
*LAG3* is highly expressed in the KIRC tissue

An analysis of the CCLE dataset revealed a significantly high expression of *LAG3* in A-704, OS-RC-2, SNU-349, KMRC-3, SLR 26, and ACHN kidney cancer cell lines (Additional file 6: Figure S4). Next, the RT-PCR results of existing kidney cancer cell lines in the laboratory found LAG3 to be highly expressed in 786-O and ACHN cell lines, which were selected for subsequent validation (Fig. 9A). The knockdown efficiency of LAG3 small interfering reagents in 786-O and ACHN cell lines by RT-PCR was validated (Fig. 9B). Subsequently, the growth curve of the siLAG3 group was significantly slower in the 786-O and ACHN cell lines than that of the control group in the CCK8 experiment (Fig. 9F). Compared with the control group, the number of cell clones in the siLAG3 group was significantly lower in 786-O and ACHN cell lines by clone experiments (Fig. 9C). The scratch experiment revealed that the siLAG3 group was less efficient at healing scratches at 24 h than the control group (Fig. 9D). Cell migration and invasion were detected to be significantly down-regulated in the siLAG3 group by transwell assay (Fig. 9E). Combined with the preliminary results of LAG3 differential expression in KIRC versus normal kidney tissues in the HPA database (Additional file 3: Figure S1), immunohistochemistry revealed the high expression of LAG3 in KIRC was further confirmed in the tissues of our patients (Fig. 10).

## Discussion

Kidney cancer constitutes the 14th most common malignancy worldwide with 431,288 new diagnosed cases and 179,368 new deaths in 2020 [3]. The major etiologies of kidney cancer include hypertension, obesity, and smoking [22]. The ICI-based combination therapy demonstrated excellent clinical efficacy in several large clinical trials and is now the first-line care standard for patients with advanced or metastatic renal cancer with a low OS at first diagnosis [23–26]. Despite the pivotal function of the PD-1/CTLA-4 axis in the treatment of RCC has greatly improved clinical outcomes compared to previous treatment options, the majority of patients with RCC did not achieve durable clinical benefits after ICI-based combination therapy [23–26]. Therefore, it is highly essential to investigate the tumor immune microenvironment and explore novel immunotherapeutic targets, and ultimately optimize systemic treatment for RCC.

In response to the immune microenvironment of renal cancer, Finke [6] and Stein [15] provided reviews on the immunology and immunobiology of renal cancer, respectively. In most solid tumors, the degree of CD8^+^ T cell infiltration was positively correlated with good prognosis for tumor patients [27]. CD8^+^ T cells exerted a direct cytotoxic effect on target cells and performed a critical role in anti-cancer immunity. However, the expression of suppressive molecules (PD-1 and CTLA-4) on the surface of CD8^+^ T cells increases in response to sustained stimulation by tumor-specific antigens, and their function decreases and eventually reaches the exhausted state, as demonstrated in multiple cancer models [13, 14, 28]. Therefore, blocking their inhibitory signaling using anti-PD1/CTLA-4 antibodies could rejuvenate exhausted CD8^+^ T cells, enhance their cytotoxicity, promote tumor cell lysis, and restrict tumor metastasis [29]. However, kidney cancer had a distinctive immune profile such that the degree of CD8^+^ T cell infiltration is positively correlated with poor prognosis, and the specific mechanism was unclear. Several hypotheses have been proposed to explain this paradoxical phenomenon. First, the activation status and virulence potential of CD8^+^ T cells were highly specific in kidney cancer, where stem-like TCF1^+^ or PD-1^+^ TIM3^−^ LAG3^−^ CD8 + T cell subsets contribute to the anti-cancer immune effect [30–32]. Second, the low density of tertiary lymphoid structures generated numerous immature DC cells, causing the infiltration of polyclonal CD8^+^ T cells that could not recognize tumor-associated antigens [11, 27, 32]. Third, the absence of tumor-specific genes, such as the relative absence of *PBRM1* mutations in highly CD8^+^ T cells RCC, which was often associated with a better prognosis [33]. Finally, specific metabolic dysregulation of CD8^+^ T cells in RCC restricted CD8^+^ T cell activation and did not recover through the PD-1 axis inhibition [34].

The high significance of CD8^+^ T cells in the immunotherapy of kidney cancer and the results of single-cell sequencing analysis (CD8 + T cells were significantly differentially expressed in KIRC and kidney tissue) can be used to construct a prognostic model of CD8^+^ T cell-associated genes to guide clinical decisions in KIRC. First, the top 10 DEGs in CD8^+^ T cells of KIRC were obtained by cluster identification in single-cell analysis, including *GZMK*, *CD27*, *CCL4L2*, *FXYD2*, *LAG3*, *RGS1*, *CST7*, *DUSP4*, *CD8A*, and *TRBV20-1*. Subsequently, the above gene expression was divided into two subtypes by cluster analysis, that is, CD8^+^ T cell-associated gene low expression (C1) and high expression (C2) subtypes. The cluster analysis results were used for grouping, and the DEGs, pathway enrichment, and mutated genes between C1 and C2 subtypes were comprehensively studied. The up-regulated genes were largely enriched for glutamate and leukotriene activity, whereas the down-regulated genes were enriched for immune-related activities. Furthermore, *VHL* and *STED2*, the most commonly mutated genes in primary KIRC, displayed remarkably high mutation rates in the C2 subtype compared to the C1 subtype, predicting a poor prognosis such as metastasis or drug resistance in the C2 subtype [35, 36]. We assessed the KIRC immune microenvironment in different subtypes and revealed that the stromal and immune scores were significantly higher in the CD8^+^ T cell-associated gene high expression subtype than in the C1 subtype, with significant differences between the two subtypes in immune infiltrating cells and immune-related molecules. Therefore, LASSO regression and Cox univariate analyses were used to construct the CD8^+^ T cell-associated risk prognostic model: RiskScore =  − 0.291858656434841*GZMK − 0.192758342489394*FXYD2 + 0.625023643446193*LAG3 + 0.161324477181591*RGS1 − 0.380169045328895*DUSP4 − 0.107221347575037*TRBV20-1, which will assist the clinicians to assess the prognosis for survival, immune status, and drug selection in patients with KIRC. Relevant studies were investigated to identify CD8^+^ T cell-related genes in kidney cancer. Genomics, radiology, and artificial intelligence modalities can be used to identify renal cancer differentiation more easily and earlier, and predict its Fuhrman grade and responsiveness to immunotherapy response, thus assisting the clinicians in defining the risk of stratification of the disease, treatment choices, follow-up strategies, and prognosis [42, 43].

Using tumor single-gene related studies as the reference [44], we performed single-gene analysis of the above genes and finally identified *LAG3* as the most valuable CD8^+^ T cell-associated gene in KIRC. Lymphocyte activating gene 3 (LAG3) or CD223 is highly expressed in different T cells, CD8^+^ T cells, CD4^+^ T cells, and Tregs, to maintain homeostasis [37]. However, persistent tumor-associated antigen stimulation causes its chronic expression, ultimately promoting T-cell exhaustion in cancers [37, 38]. Therefore, LAG3 serves as the third clinical checkpoint in case of limited or even no response in 60 to 80% of cancer patients in PD1/CTLA-4 axis immunotherapy [39]. Currently, several clinical trials are being conducted on immunotherapies targeting LAG3 in combination with PD1/CTLA-4 axis inhibitors to treat cancers including kidney cancer 40, 41. In the current study, we analyzed for the first time the distribution of LAG3 in renal cancer and renal tissue using single-cell analysis and investigated its expression, prognostic significance, immune microenvironmental relevance, and pathway enrichment using bioinformatics techniques. We confirmed that LAG3 promotes the progression and metastasis of renal cell carcinoma and is positively correlated with CD8^+^ T cells using cell phenotype studies and immunohistochemistry. Presently, immunotherapy targeting LAG3 is largely used for melanoma, pancreatic cancer, and hematological tumors, with only a few studies on renal cancer. We elucidated the function of LAG3 in KIRC. We believe our findings will provide a preliminary basis and direction for LAG3-targeted immunotherapy and even CAR-T therapy in patients with kidney cancer.

The present study had certain limitations. First, heterogeneity obtained in retrospective studies needs to be verified by conducting prospective studies. Second, we only applied the top 10 CD8^+^ T cell-associated genes to construct the risk prognosis model, which lacked comprehensiveness and extensiveness. Third, we only validated the function of LAG3 in KIRC at the in vitro level and lacked in vivo experiments, as well as the expression mechanism and CD8^+^ T cell activity warrant further exploration. In conclusion, more basic and large clinical trials are required to validate these findings (Table 1).

**Table 1:** Single-cell sequence dataset information

Dataset	Title	Number of samples	Samples
GSE121636	Single-cell sequencing of peripheral blood and tumor-infiltrating immune cells in renal clear cell carcinoma [5' RNA expression sequencing]	3 tumor-infiltrating immune cells	GSE3440844, GSE3440845, GSE3440846
GSE131685	Single-cell RNA sequencing of human kidney	2 primary kidney samples	GSE4145204, GSEE4145205

## Conclusion

The top 10 CD8^+^ T cell-associated genes were obtained by single-cell analysis, including *GZMK*, *CD27*, *CCL4L2*, *FXYD2*, *LAG3*, *RGS1*, *CST7*, *DUSP4*, *CD8A*, and *TRBV20-1*. These genes were divided into low- and high-expression subtypes by cluster analysis, and DEGs, pathway enrichment, mutant genes, and KIRC immune infiltration in different subtypes were studied. The best risk prognosis model was constructed (RiskScore =  − 0.291858656434841*GZMK − 0.192758342489394*FXYD2 + 0.625023643446193*LAG3 + 0.161324477181591*RGS1 − 0.380169045328895*DUSP4 − 0.107221347575037*TRBV20-1). Finally, the single-gene analysis identified *LAG3* as the most valuable CD8^+^ T cell-associated gene in KIRC, which was further confirmed by cell phenotype studies and immunohistochemistry.

### Supplementary Information

**Additional file 1: Table S1.** Annotated table of single cell subtypes.

**Additional file 2: Table S2.** Basic information on immunohistochemistry patients in the HPA database.

**Additional file 3: Figure S1.** Immunohistochemistry of CD8^+^ T cell-associated genes in normal renal tissue and KIRC tissue in the HPA database. (A) CD8A, (B) CD27, (C) CST7, (D) GZMK, (E) LAG3, (F) RGS1, and (G) FXYD2.

**Additional file 4: Figure S2.** Overall survival, disease-specific survival, progression-free interval, diagnostic ROC, and time-dependent ROC analysis of CD8^+^ T cell-associated genes in the TCGA–KIRC cohort. (A) CCL4L2, (B) CD8A, (C) CD27, (D) CST7, (E) DUSP4, (F) FXYD2, (G) GZMK, (H) LAG3, (I) RGS1, and (J) TRBV20-1.

**Additional file 5: Figure S3.** Correlation of *LAG3* expression with different T cell subtypes. (A) CD8 T cells. (B) Cytotoxic cells. (C) T cells. (D) T helper cells. (E) Tcm. (F) Tem. (G) TFH. (H) Tgd. (I) Th1 cells. (J) Th17 cells. (K) Th2 cells. (L) TRegs.

**Additional file 6: Figure S4.** Expression of *LAG3* in renal cancer cell lines predicted using the CCLE database.

## Data Availability

Renal epithelial cells (HK-2) and renal cancer cell lines (ACHN and 786-O) were obtained from the surgical laboratory of Zhongda Hospital, Southeast University, and were provided by Dr. Weipu Mao.
