# Supplementary material for: Single-cell combined bioinformatics analysis: construction of immune cluster and risk prognostic model in kidney renal clear cells based on CD8+ T cell-associated genes
Source: Eur J Med Res. 2024 Jan 30;29:89. doi: 10.1186/s40001-024-01689-8 (PMC10825992; doi:10.1186/s40001-024-01689-8)
Supplement: Supplementary file 1 — Additional file 1: Table S1. Annotated table of single cell subtypes. [file 40001_2024_1689_MOESM1_ESM.docx]

| **0** | **1** | **2** | **3** | **4** | **5** | **6** | **7** |
| --- | --- | --- | --- | --- | --- | --- | --- |
| MT1H | GZMK | CD52 | GSTA2 | PCK1 | GZMH | GZMB | KLRB1 |
| CD24 | CD27 | CRIP1 | PCK1 | DCXR | ANXA1 | GNLY | CD7 |
| KRT18 | CCL4L2 | IL7R | GSTA1 | MIOX | NKG7 | KLRD1 | HOPX |
| GSTP1 | FXYD2 | SLC2A3 | HPD | ADIRF | GNLY | KLRB1 | XCL1 |
| MT1G | LAG3 | RGCC | FABP1 | GLYATL1 | FGFBP2 | KLRF1 | HSPA1A |
| SPP1 | RGS1 | LTB | APOM | RIDA | KLF2 | CCL4 | XCL2 |
| CCL2 | CST7 | ANXA1 | ADIRF | ALDH6A1 | KLRG1 | SPON2 | GNLY |
| VIM | DUSP4 | GPR183 | DPEP1 | ALDOB | IL7R | FGFBP2 | KRT86 |
| SOD2 | CD8A | TRBV12-4 | RBP4 | ALDH2 | HSPA1A | AREG | HSPA6 |
| RARRES2 | TRBV20-1 | LMNA | MTRNR2L1 | RBP4 | HSPA6 | CCL3 | KRT81 |
| [Epithelial cell](javascript:;) | CD8+ T cell | B cell | [Mastocyte](javascript:;) | [Mastocyte](javascript:;) | NKT cell | NKT cell | NKT cell |
| **7** | **8** | **9** | **10** | **11** | **12** | **13** | **14** |
| KLRB1 | CST3 | S100A8 | CTSW | IL32 | IGHV5-51 | MEG3 | TUBB2B |
| CD7 | HLA-DRA | S100A9 | CD8A | FOXP3 | IGLV2-23 | POLR2J3.1 | KNG1 |
| HOPX | HLA-DPA1 | LYZ | CD52 | BATF | CD79A | MALAT1 | PVALB |
| XCL1 | HLA-DPB1 | FCN1 | GZMA | LTB | IGHA1 | NEAT1 | DEFB1 |
| HSPA1A | HLA-DQB1 | LST1 | GZMH | TIGIT | IGKV1-5 | ACSM2A | S100A2 |
| XCL2 | HLA-DRB1 | CTSS | CD8B | TNFRSF18 | IGLC2 | RNF213 | CD24 |
| GNLY | HLA-DRB5 | AIF1 | TRBV5-4 | CTLA4 | IGKV4-1 | VMP1 | CKB |
| KRT86 | C1QC | IL1B | TRBV7-9 | TNFRSF4 | IGKV3-11 | ELF3 | S100A6 |
| HSPA6 | C1QA | VCAN | RP11-291B21.2 | TBC1D4 | IGHV3-48 | SLC47A2 | PPP1R1A |
| KRT81 | C1QB | S100A12 | ITM2C | CCR8 | IGHV3-30 | UNC5CL | UMOD |
| NKT cell | B cell | [Neutrophil](javascript:;) | B cell | Treg cell | B cell | B cell | B cell |

Supplementary Table 1: Annotated table of single cell subtypes.
