# Supplementary material for: Single-cell combined bioinformatics analysis: construction of immune cluster and risk prognostic model in kidney renal clear cells based on CD8+ T cell-associated genes
Source: Eur J Med Res. 2024 Jan 30;29:89. doi: 10.1186/s40001-024-01689-8 (PMC10825992; doi:10.1186/s40001-024-01689-8)
Supplement: Supplementary file 2 — Additional file 2: Table S2. Basic information on immunohistochemistry patients in the HPA database. [file 40001_2024_1689_MOESM2_ESM.docx]

|  | **Antibody id** | **Sex** | **Age** | **Patient id** |
| --- | --- | --- | --- | --- |
| GZMK-normal | HPA063181 | Male | 61 | 1859 |
| GZMK-KIRC |  | Male | 63 | 3156 |
| CD27-normal | HPA038936 | Male | 61 | 1859 |
| CD27-KIRC |  | Male | 63 | 3156 |
| FXYD2-normal | HPA068838 | Male | 61 | 1859 |
| FXYD2-KIRC |  | Male | 63 | 3156 |
| LAG3-normal | HPA013967 | Male | 59 | 3229 |
| LAG3-KIRC |  | Male | 56 | 1752 |
| RGS1-normal | HPA074572 | Male | 61 | 1859 |
| RGS1-KIRC |  | Female | 63 | 3616 |
| CST7-normal | HPA040442 | Male | 61 | 1859 |
| CST7-KIRC |  | Male | 63 | 3156 |
| CD8A-normal | CAB075722 | Male | 61 | 1859 |
| CD8A-KIRC |  | Male | 63 | 3156 |

Supplementary Table 2: Basic information on immunohistochemistry patients in the HPA database.
