# Supplementary figures and images for: Single-cell combined bioinformatics analysis: construction of immune cluster and risk prognostic model in kidney renal clear cells based on CD8+ T cell-associated genes
Source: Eur J Med Res. 2024 Jan 30;29:89. doi: 10.1186/s40001-024-01689-8 (PMC10825992; doi:10.1186/s40001-024-01689-8)

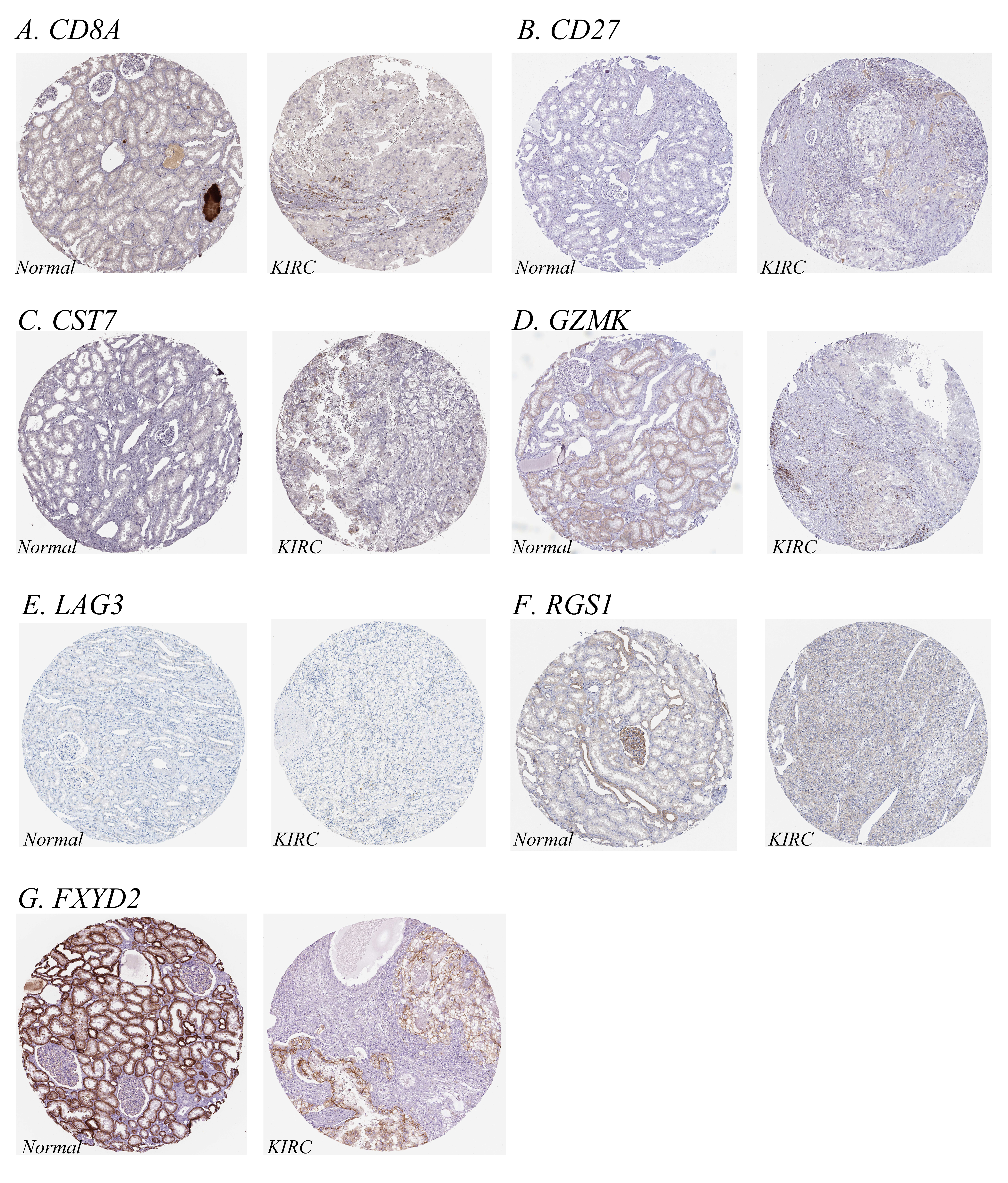

Supplement: Supplementary file 3 — Additional file 3: Figure S1. Immunohistochemistry of CD8+ T cell-associated genes in normal renal tissue and KIRC tissue in the HPA database. (A) CD8A, (B) CD27, (C) CST7, (D) GZMK, (E) LAG3, (F) RGS1, and (G) FXYD2. [file 40001_2024_1689_MOESM3_ESM.png]

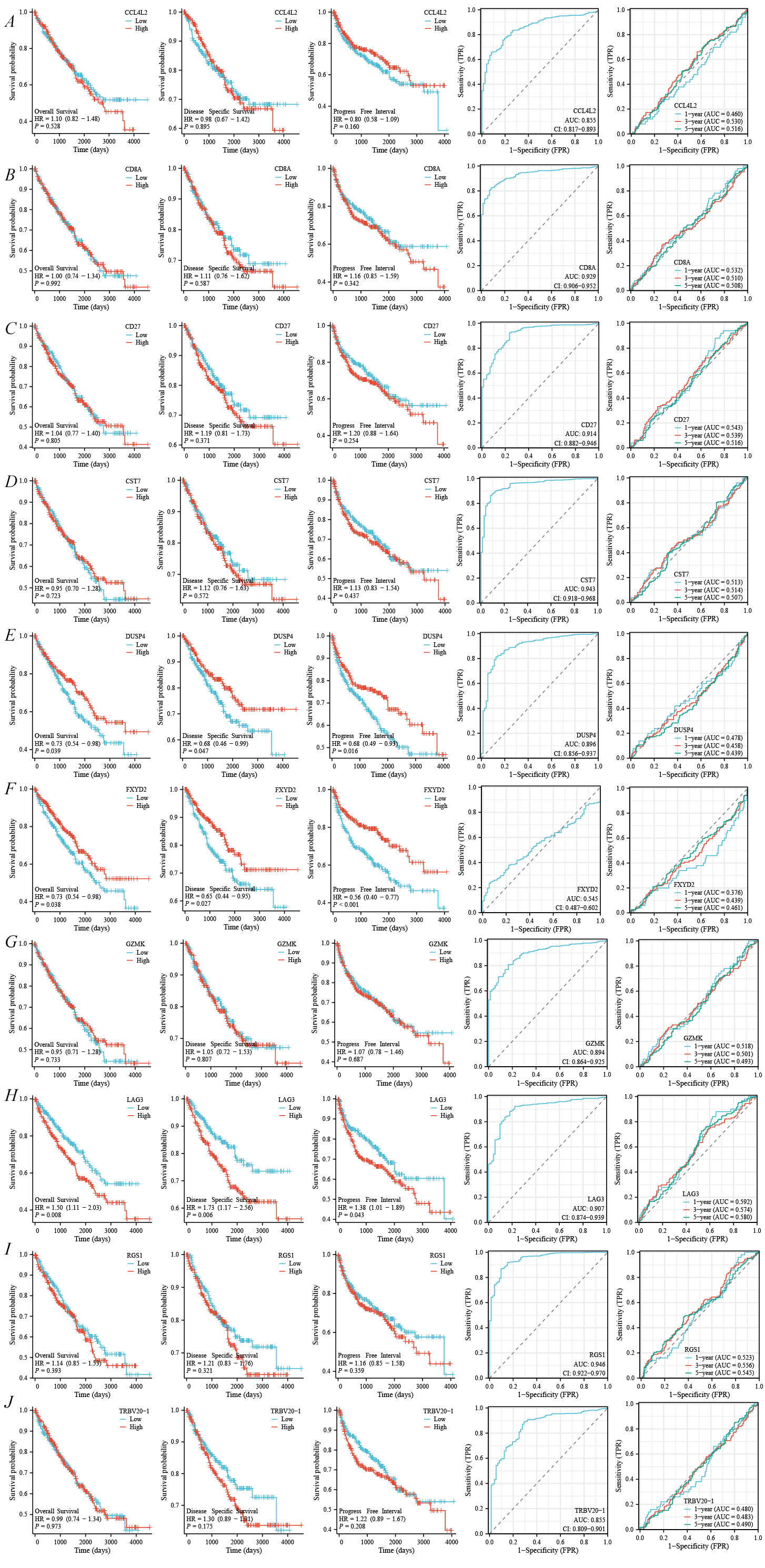

Supplement: Supplementary file 4 — Additional file 4: Figure S2. Overall survival, disease-specific survival, progression-free interval, diagnostic ROC, and time-dependent ROC analysis of CD8+ T cell-associated genes in the TCGA–KIRC cohort. (A) CCL4L2, (B) CD8A, (C) CD27, (D) CST7, (E) DUSP4, (F) FXYD2, (G) GZMK, (H) LAG3, (I) RGS1, and (J) TRBV20-1. [file 40001_2024_1689_MOESM4_ESM.tif]

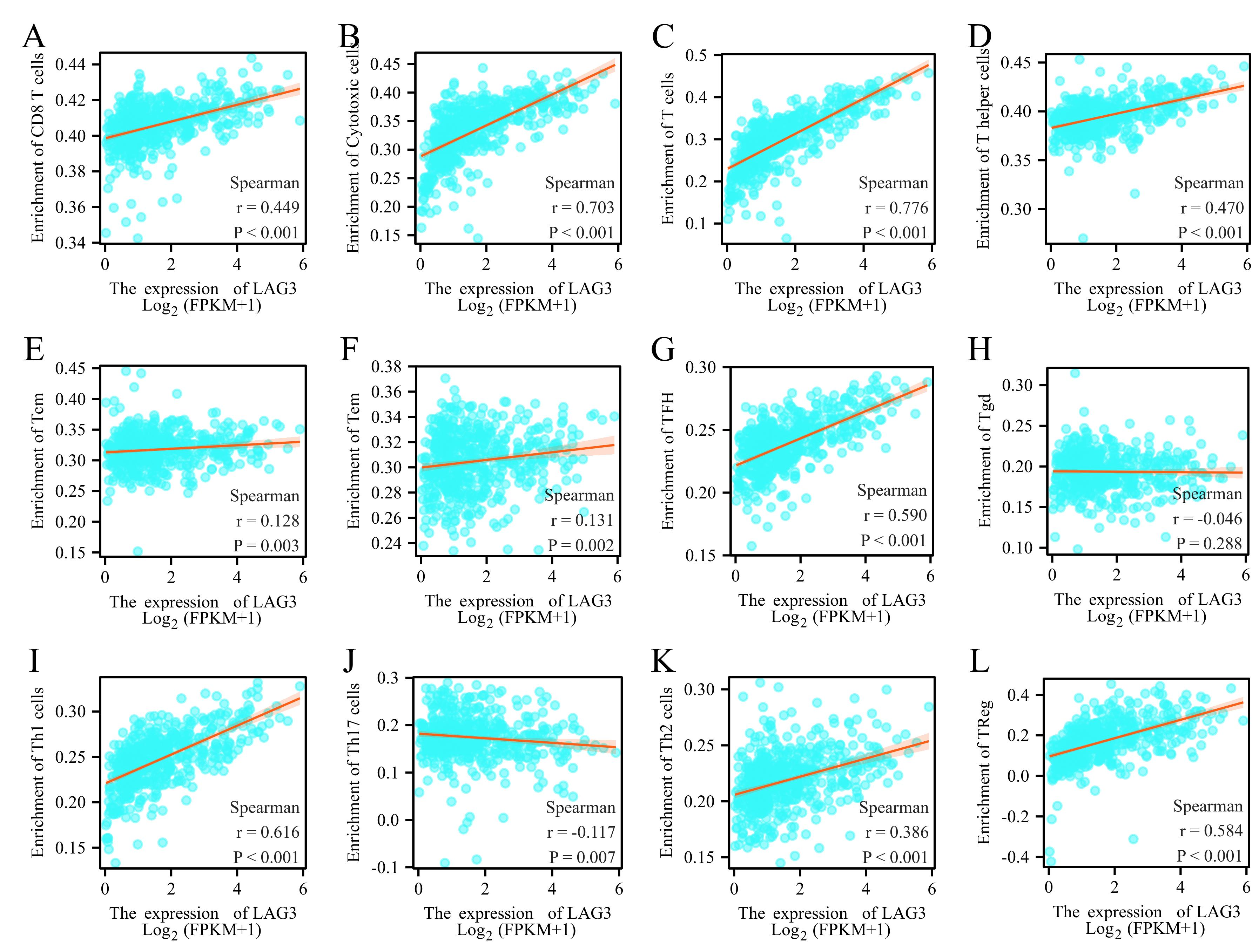

Supplement: Supplementary file 5 — Additional file 5: Figure S3. Correlation of LAG3 expression with different T cell subtypes. (A) CD8 T cells. (B) Cytotoxic cells. (C) T cells. (D) T helper cells. (E) Tcm. (F) Tem. (G) TFH. (H) Tgd. (I) Th1 cells. (J) Th17 cells. (K) Th2 cells. (L) TRegs. [file 40001_2024_1689_MOESM5_ESM.png]

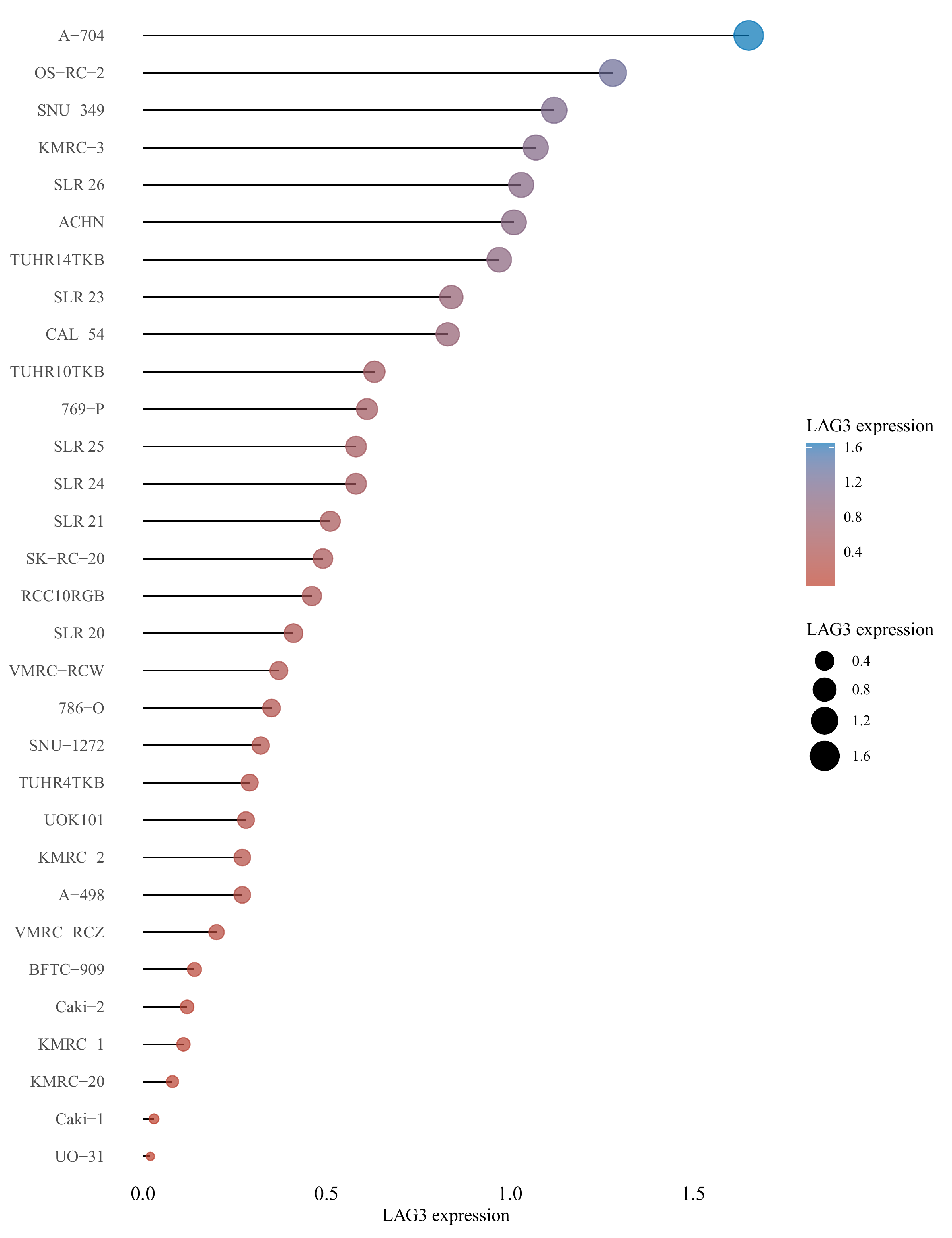

Supplement: Supplementary file 6 — Additional file 6: Figure S4. Expression of LAG3 in renal cancer cell lines predicted using the CCLE database. [file 40001_2024_1689_MOESM6_ESM.png]
